# Preliminary Study to Compare the Antimicrobial Efficacy of Aqueous and Ethanolic Ultrasound-Assisted *Aloe vera* Extracts

**DOI:** 10.3390/plants15203140

**Published:** 2026-10-14

**Authors:** Luis Cabanillas-Chirinos, Moisés Gallozzo-Cárdenas, Magali Tantaleán Pérez, María Mondéjar Barrios, David García-Cedrón

**Affiliations:** 1 Centros e Institutos de Investigación, Universidad Cesar Vallejo, Trujillo 13001, Peru; mgallozzo@ucv.edu.pe; 2 Dirección de Investigación, Universidad Cesar Vallejo, Trujillo 13001, Peru; mtantalean@ucv.edu.pe; 3 Facultad de Ciencias de la Salud, Universidad Cesar Vallejo, Trujillo 13001, Peru; mmondejar@ucv.edu.pe; 4 Facultad de Medicina, Universidad Nacional de Trujillo, Trujillo 13001, Peru; dcgarcia@unitru.edu.pe

**Keywords:** *Aloe vera*, ultrasound-assisted extraction, FTIR, antimicrobial activity, plant extracts

## Abstract

The growing threat of antimicrobial resistance has driven the search for natural alternatives, such as *Aloe vera* extracts, whose phytochemical composition and biological activity depend on the extraction solvent. This preliminary study compared the antimicrobial efficacy of aqueous and ethanolic (99%) A. vera extracts obtained by ultrasound-assisted extraction (UAE) under identical conditions, and explored their FTIR spectroscopic profiles. The extracts were characterized by FTIR and evaluated against *Staphylococcus aureus* ATCC 12493 and *Escherichia coli* ATCC 33876 using the agar well diffusion method and broth macrodilution to determine the Minimum Inhibitory Concentration (MIC) and Minimum Bactericidal Concentration (MBC). In the well diffusion assay, the ethanolic extract produced larger inhibition halos than the aqueous extract (22.3 and 20.0 mm vs. 20.6 and 17.4 mm against *S. aureus* and *E. coli*, respectively, at 200 mg/mL), and two-way ANOVA showed significant effects of extract type and microorganism (*p* < 0.001). The ethanolic extract also showed a trend toward lower mean MIC values (62.0 and 85.6 mg/mL) than the aqueous extract (90.7 and 145.0 mg/mL); however, given the high standard deviations and the limited number of replicates (*n* = 3), the MIC/MBC results are inconclusive, as no specific statistical analysis was applied to them. Likewise, the FTIR spectra showed a relatively higher intensity of bands consistent with polysaccharides (O–H and C–O–C) in the aqueous extract and of the 1600–1650 cm^−1^ band (C=O/C=C), a spectral feature potentially associated with compounds such as anthraquinones and flavonoids, in the ethanolic extract; nevertheless, owing to band overlap, the semi-quantitative nature of the comparison and the absence of complementary analyses (e.g., HPLC-MS or NMR), these results are also inconclusive regarding the specific compound classes involved. Overall, the findings indicate a trend toward higher antimicrobial activity of the ethanolic extract against the two reference strains evaluated, which should be confirmed in future studies using broth microdilution with a larger number of replicates, identification of bioactive compounds, and a broader panel of microorganisms, including resistant clinical isolates, together with cytotoxicity assays.

## 1. Introduction

The indiscriminate use of antibiotics has accelerated the emergence of resistant bacterial strains, a phenomenon that the World Health Organization considers one of the greatest threats to public health in the 21st century [1,2]. It is estimated that, without effective strategies, infections by multidrug-resistant microorganisms could cause millions of annual deaths in the coming decades [1,2]. In this context of antimicrobial resistance and the need for natural alternatives, the loss of effectiveness of standard therapies has critically intensified the vulnerability of patients to various pathologies [3]. Respiratory and dermal infections are frequently caused by multidrug-resistant bacteria, especially Gram-positive cocci such as *Staphylococcus aureus* and *Streptococcus* spp. [3,4]. These microorganisms colonize skin lesions and hinder tissue regeneration, delaying wound healing [3,4]. Faced with growing resistance to conventional antimicrobials, the need for new therapeutic alternatives has become evident, sparking increasing interest in natural products as sources of bioactive compounds (such as polyphenols and polysaccharides) capable of offering effective microbicidal action through membrane disruption or inhibition of essential proteins, without the adverse effects associated with synthetic drugs [3,5].

*Aloe vera* is a succulent plant that forms rosettes of fleshy, elongated leaves with serrated edges and a thick cuticle that allows it to retain water in arid environments [6]. Traditionally, it has been used for centuries in the folk medicine of multiple cultures to treat burns, skin cuts, mild fever, gastrointestinal disorders, immune modulation, and dermal infections [6,7]. The diversity of therapeutic effects of *A. vera* has been linked to the presence of a wide variety of metabolites, including polysaccharides, anthraquinones, flavonoids, vitamins, and enzymes [8]. Among its key secondary metabolites are polysaccharides such as glucomannans and, primarily, acemannan [9,10]. Its bioactive components include anthraquinones such as aloin, emodin, and aloe-emodin, to which part of the extract’s antimicrobial activity is attributed. These molecules can alter bacterial membrane permeability and affect essential processes such as protein synthesis [5]. Furthermore, its flavonoids and phenolic compounds act as potent free radical scavengers and actively contribute to the overall antimicrobial effect [11]. All these compounds confer upon the extract a widely documented antimicrobial activity against both Gram-positive bacteria (*S. aureus*, *Bacillus subtilis*, and *Bacillus cereus*) and Gram-negative bacteria (*Escherichia coli*, *Pseudomonas aeruginosa*, *Klebsiella pneumoniae*, and *Shigella* spp.) [12,13].

The efficacy of a plant extract depends on the extraction method and the solvent used. These factors determine the solubility and stability of phytochemicals, and therefore the yield of active compounds [11,13]. Ultrasound-assisted extraction (UAE) offers advantages over conventional techniques. The acoustic cavitation it generates breaks cell membranes and improves mass transfer [13,14]. This mechanism allows for shorter extraction times, higher yields, and the preservation of thermolabile compounds that would otherwise be degraded by the excessive heat of classical methods [13,14]. Likewise, the choice of solvent directly affects the chemical profile and biological activity of the extract by discriminating metabolites according to their affinity [15]. Thus, water extracts highly hydrophilic polar compounds such as organic acids and bioactive polysaccharides [5,11], while ethanol extracts medium-polarity compounds, facilitating the selective recovery of phenols, flavonoids, and antimicrobial anthraquinones [6,13].

Fourier transform infrared spectroscopy (FTIR) is based on the absorption of infrared radiation by molecular bonds, which vibrate at specific frequencies according to their chemical nature and environment [8,16]. This technique provides a profile of the functional groups present in a sample, making it a useful tool for the characterization of plant extracts [8,16]. In the profiling of plant extracts such as *A. vera*, FTIR stands out as a highly sensitive tool for detecting characteristic functional groups. The O–H stretching band (3200–3600 cm^−1^) indicates the presence of hydroxyl groups. These groups are characteristic of phenolic compounds, alcohols, and carbohydrates [17,18,19,20]. Likewise, the carbonyl stretching band -C=O (1600–1650 cm^−1^) is associated with the presence of conjugated carbonyls, amide bonds, and asymmetric interactions of carboxylate groups present in polysaccharides or proteins [21,22,23]. Finally, the vibration range of C-O-C bonds (1000–1300 cm^−1^) reveals the characteristic stretches of the carbohydrate skeleton and glycosidic bonds of complex plant polysaccharides, such as acemannan, alginate, or pectin [5,18]. FTIR has broad application in phytochemical profiling and quality control. It allows for rapid and non-destructive verification of botanical authenticity and the compatibility of phyto-ingredients in complex matrices. It also allows for monitoring possible degradation or structural variations induced by solvents or contaminants [2,8,20].

Despite the documented advantages of ultrasound-assisted extraction (UAE) for optimizing phytochemical yield and safeguarding the integrity of plant secondary metabolites [14], there is currently a significant knowledge gap due to the lack of comparative studies that systematically integrate FTIR characterization and antimicrobial evaluation of *A. vera* extracts obtained by UAE using different solvents [13]. Since the polarity and profile of the recovered compounds critically depend on the type of solvent, it is essential to elucidate how these variations alter the final bioactivity against clinically relevant pathogens [15,17]. Based on the above, this study was designed to answer the following question: does the extraction solvent (water vs. ethanol) significantly affect the FTIR fingerprint and antimicrobial efficacy of *A. vera* extracts obtained by UAE? To address this question, three testable hypotheses were formulated: (H1) the extraction solvent significantly modulates the FTIR fingerprint of *A. vera* extracts, with the aqueous extract being richer in bands associated with polysaccharides (O–H and C–O–C), while the ethanolic extract presents higher intensity in the carbonyl band (C=O/C=C) potentially associated with anthraquinones and flavonoids; (H2) the ethanolic extract exhibits greater antimicrobial activity than the aqueous extract against Gram-positive and Gram-negative reference strains, reflected in lower MIC/MBC values and larger inhibition halos; and (H3) the antimicrobial efficacy of the extracts depends on the type of microorganism, with both being more effective against the Gram-positive strain *S. aureus* than against the Gram-negative *E. coli*.

The choice of *A. vera* as the study material is justified for several reasons. First, it is one of the most researched medicinal plants, with more than 75 bioactive constituents and recognized broad-spectrum antimicrobial activity [8]. Second, its phytochemical composition contains polysaccharides (acemannan, glucomannans) with immunomodulatory properties and anthraquinones (aloin, emodin) with direct microbicidal activity, making it an ideal model to study the effect of the solvent on the phytochemical profile [5,9]. Third, *A. vera* is widely cultivated and commercialized in northern Peru, which creates the need to establish protocols for obtaining standardized extracts with antimicrobial activity to secure a phytotherapeutic resource for the region [6]. Finally, there is a lack of studies that systematically integrate FTIR characterization with the antimicrobial evaluation of extracts obtained by UAE using different solvents [13]. In this context, the objective of this preliminary study was to compare the effect of the extraction solvent (water vs. ethanol) on the phytochemical profile and antimicrobial efficacy of *A. vera* extracts. Ultrasound-assisted extraction (UAE) and FTIR spectroscopy were employed as methodological tools to achieve this objective.

The novelty of this study lies in the systematic comparison of aqueous and ethanolic *A. vera* extracts obtained under identical UAE conditions, to determine the effect of the solvent on the FTIR profile and antimicrobial activity. As a secondary contribution, the utility of FTIR-ATR as a quality control tool for the standardization of phytopharmaceutical extracts is explored.

## 2. Results

### 2.1. Extraction Yield

The extraction yield of *A. vera* extracts was evaluated using distilled water and 99% ethanol as solvents, maintaining constant ultrasound-assisted extraction (UAE) conditions. For each extraction, 50.00 g of dried leaf powder was used. The results, summarized in Table 1, show that the aqueous extract presented an average yield of 14.1 ± 0.3% (*w*/*w*), while the ethanolic extract reached a value of 12.8 ± 0.3% (*w*/*w*). Although the yield of the aqueous extract was slightly higher, the difference between the two solvents was modest (approximately 1.3 percentage points). The low standard deviation in both cases (<0.4%) indicates good reproducibility of the ultrasound extraction process under the conditions tested, which may facilitate the standardization of *A. vera* extracts in future studies.

### 2.2. Comparative Analysis of FTIR Spectra

Figure 1 presents the FTIR spectra of the aqueous and ethanolic *A. vera* extracts obtained by ultrasound-assisted extraction (UAE), recorded in the range of 4000 to 500 cm^−1^ [13,19]. Both spectra showed the characteristic bands of the functional groups present in the plant’s phytochemical matrix. The broad and intense band in the 3300–3400 cm^−1^ region, attributed to O–H stretching of alcohols, phenols, and polysaccharides [9,17,22], was notably more intense in the aqueous extract. In the 2800–2950 cm^−1^ region, corresponding to C–H stretching of aliphatic groups (alkanes and lipids) [6,19], similar intensity was observed in both extracts. The band located at 1600–1650 cm^−1^, of potential interest for antimicrobial activity, may be attributed to C=O stretching of conjugated carbonyls and/or C=C bonds of unsaturated compounds such as flavonoids and anthraquinones [11,15,24], and showed higher relative intensity in the ethanolic extract. However, given that the exact composition of the extracts is unknown, this band cannot be exclusively assigned to a single functional group, and the intensity comparison should be interpreted as semi-quantitative. Finally, the 1000–1150 cm^−1^ region, corresponding to C–O–C stretching of glycosidic bonds of complex plant polysaccharides [9,18,25], presented higher intensity in the aqueous extract. These spectral differences suggest that the choice of solvent influences the phytochemical profile of extracts obtained by UAE: the aqueous extract showed bands consistent with polar polysaccharides, while the ethanolic extract showed spectral features potentially associated with phenolic compounds and anthraquinones [9,13,15]. The stock solutions had concentrations of 870 mg/mL (aqueous) and 850 mg/mL (ethanolic), with a difference of only 2.3%, and the spectra were normalized to allow for a semi-quantitative comparison of the phytochemical profiles.

Table 2 presents the qualitative differences observed between the aqueous and ethanolic *A. vera* extracts, identified from the analysis of the FTIR spectra, which allowed for the recognition of the main functional groups present in each. The spectra showed differentiated phytochemical profiles according to the extraction solvent, suggesting that the choice of solvent influences the composition of recovered metabolites. The aqueous extract was characterized by a marked predominance of bands associated with polysaccharides (O–H at 3300–3400 cm^−1^ and C–O–C at 1000–1150 cm^−1^), consistent with the presence of polysaccharides such as acemannan and glucomannans, major structural components of the *A. vera* gel [9,17]. In contrast, the ethanolic extract showed a higher relative intensity in the C=O/C=C band (1600–1650 cm^−1^), a spectral feature potentially associated with anthraquinones (aloin, emodin, aloe-emodin) and flavonoids, compounds with recognized direct antimicrobial activity [8,26]. Overall, the FTIR analysis suggests relative trends: the ethanolic extract showed bands consistent with secondary metabolites of medium polarity, while the aqueous extract showed bands consistent with structural polysaccharides; these trends require confirmation by complementary techniques such as HPLC-MS or NMR.

### 2.3. Antimicrobial Susceptibility Tests

#### 2.3.1. Quantitative Test: Broth Macrodilution Method

The antimicrobial activity of the aqueous and ethanolic *A. vera* extracts was evaluated against the reference strains *S. aureus* ATCC 12493 (Gram-positive) and *E. coli* ATCC 33876 (Gram-negative). The results in Table 3 indicate that the ethanolic extract presented lower mean MIC and MBC values than the aqueous extract against both evaluated strains; however, given the high standard deviations and the limited number of replicates (*n* = 3), these differences should be interpreted as a trend rather than as conclusive evidence, since no specific statistical analysis was applied to the MIC/MBC data. Likewise, both extracts showed a trend toward lower mean MIC values against the Gram-positive strain *S. aureus* compared to the Gram-negative *E. coli*. The controls showed the expected performance: the positive growth control exhibited visible turbidity, the sterility control remained clear, and the solvent control (DMSO 50% *v*/*v*) did not inhibit bacterial growth. The MIC values were determined as the lowest concentration that inhibited visible growth, and the MBC as the lowest concentration that killed ≥99.9% of the initial inoculum. The concentrations tested in the twofold dilution series were 870, 435, 217.5, 108.75, 54.38, 27.19, 13.59, 6.80, and 3.40 mg/mL for the aqueous extract, and 850, 425, 212.5, 106.25, 53.13, 26.56, 13.28, 6.64, and 3.32 mg/mL for the ethanolic extract. The MIC/MBC values reported in Table 3 correspond to these concentrations.

#### 2.3.2. Qualitative Test: Agar Well Diffusion Method

The antimicrobial activity of the aqueous and ethanolic *A. vera* extracts was evaluated using the well diffusion method against the reference strains *S. aureus* ATCC 12493 (Gram-positive) and *E. coli* ATCC 33876 (Gram-negative). The inhibition halos obtained for each concentration are summarized in Table 4. The results showed a concentration-dependent behavior for both extracts, with a progressive decrease in inhibition diameters as the extract concentration was reduced. The ethanolic extract produced larger inhibition halos than the aqueous extract at all active concentrations (200, 100, and 50 mg/mL). It can also be observed that at concentrations of 25 mg/mL and 12.5 mg/mL, neither extract showed inhibitory activity. Positive and negative controls were included in all assays to validate the well diffusion method. The positive control produced inhibition halos of 30.0 ± 0.6 mm against *S. aureus* (vancomycin 30 µg/well) and 25.0 ± 0.6 mm against *E. coli* (ciprofloxacin 5 µg/well), confirming the sensitivity of both reference strains and the validity of the assay (*n* = 10). The negative control (DMSO) did not produce an inhibition halo in any case (0.0 mm), ruling out any antimicrobial effect attributable to the solvent used to resuspend the extracts.

#### 2.3.3. Statistical Analysis

Table 5 summarizes the results of the statistical analysis using two-way ANOVA, which evaluated the effects of extract type (aqueous vs. ethanolic), microorganism (*S. aureus* vs. *E. coli*), and their interaction on inhibition diameters at concentrations that showed antimicrobial activity. At the 200 mg/mL concentration, highly significant effects were observed for both extract and microorganism (*p* < 0.001 in both cases); in contrast, at the 100 mg/mL concentration, in addition to the significant main effects of extract (*p* < 0.001) and microorganism (*p* < 0.001), a highly significant interaction was detected (*p* < 0.001). For the 50 mg/mL concentration, the aqueous extract showed no activity (halos = 0); therefore, only the ethanolic extract was compared between strains using Student’s *t*-test (*p* = 0.112).

## 3. Discussion

The extraction yields obtained in the present study (14.13 ± 0.31% for the aqueous extract and 12.80 ± 0.32% for the ethanolic extract) are within the range reported in previous research that used ultrasound-assisted extraction (UAE) on *A. vera*. The study by Behaiyn et al. (2023) optimized the extraction of aloins from *A. vera* leaf skin using sonication with 80% ethanol, reporting an extraction yield of 9.43% [27]. In contrast, Dehghan et al. (2025) reported a significantly higher extraction yield of 23.230 ± 0.007% for *A. vera* rind using the same UAE technology, but employing 80% ethanol as the extraction solvent [13]. This difference in yield shows that the combination of water and an organic solvent noticeably improves the dielectric constant and solubility of the mixture. Likewise, Akbari et al. (2021) achieved a maximum yield of 36.17% using microwave-assisted extraction (MAE) of *A. vera* leaves with an optimized mixture of 43.38% ethanol, demonstrating that intermediate-polarity solutions facilitate both the cellular swelling of the mucilaginous matrix by water action and the desorption and selective solubilization of phytochemicals by the organic alcohol [11]. Other authors have documented considerably higher yields for *A. vera* gel extracts using UAE, with values of 44.02% for ethanol extraction and 52.14% for water extraction (followed by ethanol precipitation) [28]. The slight superiority of the aqueous yield (14.13%) over the ethanolic one (12.80%) observed in our study contrasts with what was reported by Mbajiuka et al. (2014), who consider ethanol as the best solvent for extracting compounds from *A. vera* [29]. This discrepancy can be attributed to the high hydrophilicity of the gel, whose polysaccharide content (glucomannans and acemannan) represents a significant fraction of the dry weight and is efficiently extracted by water. The higher yield of the aqueous extract (14.13 ± 0.31%) compared to the ethanolic one (12.80 ± 0.32%) could be related to the chemical composition of *A. vera* and the different affinity of its components for each solvent. Water favors the extraction of hydrophilic compounds, particularly polysaccharides such as acemannan and glucomannans, which can increase the total mass recovered [9,17]. In contrast, ethanol has a higher affinity for intermediate-polarity compounds, such as anthraquinones, flavonoids, and other metabolites associated with antimicrobial activity [8,13]. These results highlight the importance of selecting the extraction solvent based on the objective, whether to maximize the overall yield or favor the recovery of compounds with potential antimicrobial activity: likewise, the low standard deviation in both cases (<0.35%) indicates good reproducibility of the UAE process, in agreement with Aung and Das (2025), who highlight that acoustic cavitation improves mass transfer and extraction efficiency in reduced times [14].

The FTIR spectra obtained in the present study revealed notable qualitative differences between the aqueous and ethanolic *A. vera* extracts, which reflect the selectivity of each solvent on the functional groups present in the plant matrix. The broad and intense band in the 3300–3400 cm^−1^ region, attributed to O–H stretching of alcohols, phenols, and polysaccharides, was markedly more intense in the aqueous extract, which coincides with what was reported by Elferjane et al. (2023), who found that extracts obtained with high-polarity solvents presented greater intensity in this region, associated with the abundant presence of structural polysaccharides such as acemannan and glucomannans [28]. This predominance has also been documented by Pandit et al. (2017), who identified a high-intensity vibration of the polar hydroxyl groups of the gel at 3417 and 3401 cm^−1^ for different plant genotypes [30], and by Ray et al. (2013), who assigned this behavior to the phenolic and polar carbohydrate stretching that establishes dense hydrogen bond networks [17]. In contrast, the ethanolic extract showed higher relative intensity in the 1600–1650 cm^−1^ band, which may correspond to C=O stretching of conjugated carbonyls and/or C=C bonds, spectral features potentially associated with anthraquinones (aloin, emodin, aloe-emodin) and flavonoids, which is consistent with the expected affinity of this medium-polarity solvent for concentrating lipophilic phytochemicals. This spectral difference coincides with the findings of Alharthi et al. (2026), who through FTIR and HPLC analysis demonstrated that organic solvents such as ethanol are more effective for extracting phenolic compounds and anthraquinones from *A. vera* [8], and is in line with Dehghan et al. (2025) and Akbari et al. (2021), who identified a prominent carbonyl peak at 1727 cm^−1^ (C=O) and bands at 1617 cm^−1^ associated with anthraquinone carbonyls from leaf rind using ultrasound-assisted extraction with 80% ethanol [13] and in optimized hydroethanolic extracts [11]. In contrast, in purely aqueous extracts such as those reported by El-Ebidy et al. (2026), the band in the 1639 cm^−1^ region is usually associated mainly with the bending of free water (H–O–H) or amide I-type vibrations, showing a lower contribution of bioactive phenolic carbonyls [12]. The 1000–1150 cm^−1^ region, corresponding to C–O–C stretching of glycosidic bonds and carbohydrates, presented higher intensity in the aqueous extract, which is consistent with the expected efficiency of water for recovering high-molecular-weight polysaccharides; this spectral feature may reflect the preferential dissolution of polymers containing β-1,4 glycosidic bonds, such as the acemannan polymeric skeleton, a behavior analogous to the spectrum of the purified polysaccharide ABPA7 studied by Guo et al. [9] and to the alginate and *A. vera* composite matrices reported by Chimwai et al. [23] and Pereira et al. [22], where the intense bands in the 1030 to 1050 cm^−1^ range describe the characteristic fingerprint of the plant’s soluble carbohydrate polymers. The similarity in the intensity of the C–H band (2800–2950 cm^−1^) in both extracts suggests a comparable presence of lipid and aliphatic compounds, regardless of the solvent employed. Altogether, these spectral differences are consistent with what has been reported in the literature, where it has been established that the choice of solvent influences the phytochemical profile of *A. vera* extracts, with water being more suitable for the extraction of polar polysaccharides and ethanol for the selective recovery of secondary metabolites with direct antimicrobial activity.

When comparatively analyzing the phytochemical profiles described in Table 2, it can be observed how the selectivity of the extraction solvent modulates the relative abundance of functional groups and the spectroscopic fingerprint of the *A. vera* extracts obtained by ultrasound-assisted extraction (UAE). The marked predominance of the O–H stretching band (3300–3400 cm^−1^) and the C–O–C stretching vibration (1000–1150 cm^−1^) in the aqueous extract suggests a preferential dissolution of complex hydrophilic carbohydrate polymers such as acemannan and glucomannans, which constitute the majority structural fraction of the plant gel [17,22]. This relative abundance is highly congruent with the profiles reported by Jangber et al. (2026), who characterized *Aloe barbadensis* extracts identifying hydroxyl and ether/alcohol peaks, very prominent at 3373.2 and 1058.6 cm^−1^ [19], and by Mondal et al. (2026), who associated similar signals in hydrogels with the high density of polar groups of the aloe carbohydrate fractions [25]. In contrast, the ethanolic extract exhibits higher relative superior intensity in the carbonyl C=O/C=C band (1600–1650 cm^−1^), which may correspond to the stretching of conjugated carbonyls and C=C bonds, spectral features potentially associated with anthraquinones (aloin, emodin, aloe-emodin) and flavonoids. This spectral behavior is consistent with the notion that organic solvents of medium polarity recover secondary metabolites of phenolic and lipophilic character more efficiently. This is in line with the FTIR characterizations of Dehghan et al. (2025), who reported prominent C=O peaks in the leaf rind at 1727.87 and 1617.95 cm^−1^ selectively associated with anthraquinones [13], as well as with the findings of Alharthi et al. (2026) and Farooq et al. (2022), where the response of phenolic carbonyl bands increases proportionally with the use of organic extracting phases [8,24]. It is important to acknowledge that the interpretation of the FTIR spectra is limited by the intrinsic complexity of the extracts. The absorption around 1640 cm^−1^, for instance, could arise from either C=O or C=C stretching vibrations, and without complementary techniques such as HPLC-MS or NMR, a definitive assignment is not possible. Similarly, the comparison of band intensities between the aqueous and ethanolic extracts provides only a semi-quantitative indication of relative compositional differences, as the exact concentration of each contributing compound is unknown. Therefore, the spectral differences observed should be interpreted as trends rather than as conclusive evidence of specific compound classes.

The antimicrobial mechanisms proposed for the *A. vera* extracts are based on the literature on compounds with similar functional groups. Anthraquinones (aloin, emodin, aloe-emodin) act through multiple pathways. First, they intercalate into the lipid bilayer and alter its fluidity, causing leakage of intracellular material and lysis [8,13,26]. Second, they block the A site of the ribosome and prevent protein synthesis [26]. Third, they inhibit topoisomerase II and helicase, affecting DNA replication [13]. Fourth, they interfere with solute transport in the plasma membrane [8,17]. Finally, their hydroxyl groups chelate Mg^2+^ and Ca^2+^ ions essential for the cell wall [17,26]. On the other hand, flavonoids exert their action through: (1) inhibition of nucleic acid synthesis: flavonoids inhibit DNA gyrase and RNA polymerase [24]; (2) disruption of the cytoplasmic membrane: lipophilic flavonoids intercalate into the lipid bilayer, altering its integrity [26]; and (3) inhibition of cellular respiration: flavonoids inhibit NADH-cytochrome c reductase and ATP synthase, altering energy production [17]. Polysaccharides (acemannan, glucomannans) act through: (1) modulation of the immune response: acemannan stimulates cytokine production and macrophage activation [9]; (2) formation of a physical barrier: high-molecular-weight polysaccharides form a hydrated layer that prevents bacterial adhesion [5]; and (3) bacteriostatic activity: polysaccharides interfere with the biosynthesis of bacterial cell wall components [5,9]. It is important to emphasize that these mechanisms are proposed based on the literature and have not been experimentally validated in this study; their confirmation would require specific assays such as electron microscopy, release of intracellular material (260 nm), enzyme inhibition, and synergy studies.

The antimicrobial activity quantified through the Minimum Inhibitory Concentration (MIC) and Minimum Bactericidal Concentration (MBC), whose results are summarized in Table 3, suggested a trend toward greater activity of the ethanolic extract compared with the aqueous one against the strains of *S. aureus* ATCC 12493 and *E. coli* ATCC 33876. These findings are consistent with those reported by Dehghan et al. (2025), who found that *A. vera* rind extracts obtained by ultrasound with 80% ethanol presented MIC values ranging between 6.25 and 25 mg/mL and MBC between 12.5 and 50 mg/mL [13], although in ranges lower than those of our study, which could be attributed to differences in the plant part used (rind vs. whole leaf) or in the extraction conditions. Likewise, a study with clinical isolates of *S. aureus* reported a mean MIC of 94 ± 41.23 mg/mL for *A. vera* leaf extracts [3], a value that approximates that of our aqueous extract (90.7 mg/mL), while our ethanolic extract showed a lower mean value (62.0 mg/mL). The trend toward greater efficacy of the ethanolic extract could be related to its phytochemical profile, possibly enriched in anthraquinones and flavonoids, as tentatively suggested by FTIR (Table 2), compounds that possess a structure similar to tetracyclines and have been reported to act by blocking the A site of the bacterial ribosome, inhibiting protein synthesis [26]. It is important to contextualize that the MIC and MBC values obtained in this study are consistent with what has been reported for crude extracts of *A. vera* and other medicinal plants. For example, Dehghan et al. (2025) reported MIC values between 6.25 and 25 mg/mL for *A. vera* rind extracts obtained with 80% ethanol [13], while Kshash and Ahmed (2025) reported a mean MIC of 94 ± 41.23 mg/mL for *A. vera* leaf extracts against clinical isolates of *S. aureus* [3]. Furthermore, the antimicrobial activity of crude extracts is not directly comparable to that of purified antibiotics, as the former contain relatively low concentrations of individual bioactive compounds. Nevertheless, the advantage of crude extracts lies in their possible synergistic effect and their ability to act through multiple simultaneous mechanisms, which could hinder the development of bacterial resistance; however, this aspect was not evaluated in the present study.

The results of the well diffusion test, presented in Table 4, corroborate the concentration-dependent antimicrobial activity of both extracts. At the 200 mg/mL concentration, the ethanolic extract produced inhibition halos of 22.3 ± 0.5 mm against *S. aureus* and 20.0 ± 0.4 mm against *E. coli*, significantly surpassing the aqueous extract (20.6 ± 0.4 mm and 17.4 ± 0.5 mm, respectively). This difference was maintained at 100 mg/mL, where the ethanolic extract showed halos of 14.3 ± 0.5 mm and 13.8 ± 0.6 mm, compared to 13.6 ± 0.5 mm and 11.6 ± 0.6 mm for the aqueous one. This behavior is consistent with previous studies that have documented higher antibacterial activity of ethanolic *A. vera* extracts compared to aqueous ones [29], possibly attributable to the greater extraction of phenolic compounds and anthraquinones by ethanol. The absence of activity at 25 mg/mL and 12.5 mg/mL for both extracts suggests the existence of a threshold concentration below which the bioactive compounds do not reach the effective dose necessary to inhibit bacterial growth. In the MIC and MBC assays, however, it is important to acknowledge that the values showed large standard deviations (e.g., 90.7 ± 31.4 mg/mL for the aqueous extract against *S. aureus*). This variability is common in antimicrobial assays of crude plant extracts, where the complexity of the phytochemical mixture, the visual determination of the endpoint, and possible adsorption of compounds to the tube walls can introduce heterogeneity. Despite this variability, the ethanolic extract showed lower mean MIC and MBC values than the aqueous extract for both strains, suggesting a trend that was not tested statistically. Nevertheless, the large standard deviations limit the statistical power to detect significant differences between extracts. Future studies should increase the number of replicates (*n* > 3) and employ more precise quantitative methods, such as microdilution in 96-well plates with spectrophotometric reading, to reduce variability and strengthen the statistical conclusions. It is important to highlight that both extracts exhibited larger inhibition halos (at 200 and 100 mg/mL) and a trend toward lower MIC values against the Gram-positive strain *S. aureus* compared to the Gram-negative *E. coli*, a difference that may be attributed to the cell envelope structure: Gram-positive bacteria possess a more accessible peptidoglycan layer, while Gram-negative bacteria present an additional outer membrane that acts as a permeability barrier [3,5], limiting the diffusion of antimicrobial compounds into the cell interior.

Prior to the application of the statistical analysis, the assumptions of normality and homogeneity of variances were verified to guarantee the validity of the two-way ANOVA. The Shapiro–Wilk test confirmed the normal distribution of the data in the groups with activity (halos > 0), while Levene’s test evidenced heterogeneous variances when groups with halos = 0 (zero variance) were included. For this reason, the concentrations of 25 and 12.5 mg/mL (both extracts) and 50 mg/mL of the aqueous extract, which did not present inhibitory activity, were excluded from the parametric analysis and reported descriptively. The two-way ANOVA applied to the 200 and 100 mg/mL concentrations (Table 5) revealed highly significant effects of the type of extract (*p* < 0.001) and the microorganism (*p* < 0.001) at both concentrations. At 200 mg/mL, the interaction between both factors was not significant (*p* = 0.138), indicating that the larger inhibition halos produced by the ethanolic extract compared with the aqueous one were consistent for both bacterial strains. In contrast, at 100 mg/mL, a highly significant interaction was detected (*p* < 0.001), suggesting that the difference between the extracts depends on the microorganism evaluated: against *S. aureus*, the advantage of the ethanolic extract was modest (14.3 vs. 13.6 mm), while against *E. coli*, the difference was more pronounced (13.8 vs. 11.6 mm). This finding suggests that the advantage of the ethanolic extract may be more pronounced against the Gram-negative strain at intermediate concentrations, possibly because compounds extracted by ethanol, potentially phenolic compounds and anthraquinones, may penetrate the outer membrane barrier of Gram-negative bacteria more effectively when a critical concentration is reached. For the 50 mg/mL concentration, the aqueous extract showed no activity, so only the ethanolic extract was evaluated using Student’s *t*-test, which revealed no significant differences between both strains (*p* = 0.112). Overall, these results indicate that the ethanolic *A. vera* extract obtained by ultrasound-assisted extraction produced significantly larger inhibition halos than the aqueous extract against the two reference strains evaluated, especially against the Gram-negative strain *E. coli* ATCC 33876, and that its efficacy is modulated by concentration and microorganism type. It is important to note that the antimicrobial evaluation was limited to two reference strains: *S. aureus* ATCC 12493, as a representative model of Gram-positive bacteria, and *E. coli* ATCC 33876, as a representative model of Gram-negative bacteria. Both strains were selected for their clinical relevance in respiratory and dermal infections, as well as for their standardized use in antimicrobial susceptibility testing [3,4,31]. However, it is important to clarify that these are reference strains commonly used in susceptibility testing and are not necessarily representative of multidrug-resistant clinical isolates. While the study addresses the broader context of antimicrobial resistance, the evaluation of activity against clinically resistant strains was beyond the scope of this work. Therefore, these results cannot be extrapolated to a broad antimicrobial spectrum or to resistant clinical strains. Future studies should include clinical isolates with characterized resistance profiles (e.g., methicillin-resistant *S. aureus*, extended-spectrum β-lactamase-producing *E. coli*) to determine the true potential of *A. vera* extracts against resistant pathogens.

Compared to previous studies that have evaluated aqueous and ethanolic *A. vera* extracts [8,13,17,28], this work provides several novel contributions. First, the systematic comparison of both solvents under similar conditions allows for understanding the effect of the solvent on the phytochemical profile and bioactivity, eliminating confounding variables present in studies that used different extraction methods for each solvent. Second, the integration of FTIR characterization with antimicrobial evaluation in the same experimental design allows for hypothetically establishing a correlation between the identified functional groups and the observed biological activity, which has not been systematically addressed in the previous literature. Third, the use of FTIR as a complementary quality control tool could offer a rapid, non-destructive, and reproducible method for the standardization of phytopharmaceutical extracts, which could be relevant to the pharmaceutical and food industries. Finally, this study was conducted with *A. vera* cultivated in northern Peru, which provides information on the regional variability of phytochemical composition and antimicrobial activity, an aspect little explored in the literature.

Given the preliminary nature of these findings, the present study has some limitations that must be considered when interpreting the results. First, the phytochemical characterization was based exclusively on FTIR spectroscopy, a technique that provides qualitative information on the functional groups present but does not allow for the specific identification or quantification of the individual compounds responsible for the antimicrobial activity. The absence of chromatographic techniques coupled to mass spectrometry (HPLC-MS or GC-MS) prevents establishing a direct correlation between specific compounds (such as aloin, emodin, or particular flavonoids) and the observed biological efficacy. Second, the study was conducted only under in vitro conditions against reference bacterial strains, without evaluating cytotoxicity, biocompatibility, or in vivo efficacy of the extracts. No cell viability assays in human cell lines, hemolysis tests, or dermal irritation assays were performed, which prevents confirming the safety of the extracts for therapeutic applications. Third, the antimicrobial evaluation was restricted to two reference strains (*S. aureus* ATCC 12493 and *E. coli* ATCC 33876), which limits the generalization of the results to other bacterial species or clinical strains with acquired resistance mechanisms. While these strains are representative of the Gram-positive and Gram-negative groups and are standardized for susceptibility testing [31,32], this remains a limitation. Another limitation of the study is that the stock solutions of the aqueous and ethanolic extracts presented slightly different concentrations (870 mg/mL and 850 mg/mL, respectively), with a difference of approximately 2.3%. Although this difference is small and a constant volume of each solution was deposited on the ATR crystal with controlled pressure, a minor influence on the absolute intensity of the bands cannot be ruled out. Therefore, the comparison of intensities should be interpreted as semi-quantitative, and the conclusions derived from it refer to relative trends in phytochemical composition rather than to absolute concentrations of specific compounds. Future research should aim to overcome these limitations by identifying and quantifying specific compounds using chromatographic techniques, as well as evaluating the extracts against a broader panel of microorganisms, including bacteria and fungi of clinical relevance, such as *P. aeruginosa*, *K. pneumoniae*, *Candida albicans*, and clinical isolates, to determine the true spectrum of action of *A. vera* extracts. Likewise, the performance of cytotoxicity and biocompatibility studies using human cell lines (e.g., HaCaT keratinocytes, HDF fibroblasts) and hemolysis assays, following ISO 10993 guidelines [33], is recommended to guarantee their safety for any therapeutic application.

Overall, the results provided only partial and preliminary support for the three hypotheses. H1 was tentatively supported by the differences observed in the FTIR spectra between the aqueous and ethanolic extracts, particularly in the O–H, C=O/C=C, and C–O–C regions; however, these differences are semi-quantitative and inconclusive until confirmed by complementary techniques such as HPLC-MS or NMR. H2 was supported by the significantly larger inhibition halos produced by the ethanolic extract, whereas the lower MIC/MBC values showed only a non-conclusive trend owing to their high variability. H3 was supported by the well diffusion results at 200 and 100 mg/mL and by the lower mean MIC values against the Gram-positive strain *S. aureus* than against the Gram-negative *E. coli*. These preliminary findings suggest that the extraction solvent influences both the phytochemical profile and the antimicrobial potential of *A. vera* extracts, but they should be confirmed in future studies with a larger number of replicates and more precise analytical and microbiological methods.

Based on the findings of this study and the reviewed literature, some considerations can be outlined for future research on *A. vera* extracts. First, regarding solvent selection, the use of 99% ethanol could be considered for obtaining extracts intended for further antimicrobial evaluation, given the larger inhibition halos observed against the two reference strains and the spectral features potentially associated with phenolic compounds such as anthraquinones and flavonoids [8,13,17,28]; conversely, distilled water may be more suitable when the objective is to obtain extracts rich in polysaccharides [9,17]. Second, regarding process standardization, establishing ultrasound-assisted extraction protocols with fixed parameters, such as a frequency of 40 kHz, power of 480 W, 90 min, 50 °C, and a solid:solvent ratio of 1:5 *w*/*v*, would help improve the reproducibility of the phytochemical profile and antimicrobial activity between batches [13,14,27,28]. Third, FTIR analysis could be explored as a rapid and non-destructive complementary tool to monitor the consistency of the extracts, using the bands at 3300–3400 cm^−1^ (O–H), 1600–1650 cm^−1^ (C=O/C=C), and 1000–1150 cm^−1^ (C–O–C) as tentative spectral markers, although it should be complemented by chromatographic techniques for compound identification [8,17,22]. Finally, any potential pharmaceutical, topical, or therapeutic application of these extracts would first require confirmation of their antimicrobial activity using more precise methods and a larger number of replicates, evaluation against a broader panel of microorganisms including resistant clinical isolates, and cytotoxicity, biocompatibility, and in vivo studies to establish their safety and efficacy [6,31,32].

## 4. Materials and Methods

### 4.1. Plant Material and Sample Preparation

The plant material consisted of fresh leaves of *Aloe vera* (L.) collected from a natural growth area in the Trujillo region, Peru (coordinates: 8° 06′ 27.8” S, 79° 02′ 52.1” W). Healthy plants, free of pests and at intermediate maturity (basal leaves), were selected. Botanical identification was performed by the researchers themselves, who are biologists with professional training in plant taxonomy, based on the distinctive morphological characteristics of the species *A. vera* (L.), following standard taxonomic keys for the Asphodelaceae family. It should be noted that no voucher specimen was deposited in a botanical collection, which is recognized as a limitation of the study. However, *A. vera* is a widely cultivated species, morphologically well-characterized and easily identifiable, which reduces the risk of taxonomic error.

The leaves were washed with distilled water to remove impurities, then the spines were removed, leaving the majority of the leaf (rind, gum, and pulp) for obtaining the extract. The plant material was fragmented into small pieces before being dried in an oven at 40–45 °C for 72 h until constant weight was reached. This drying temperature was selected to preserve the bioactive compounds, as it has been demonstrated that drying at 40 °C allows for the preservation of the immunomodulatory, anti-inflammatory, and antibacterial properties of *A. vera*, and that the functional properties of the gel present a maximum at this temperature [34,35]. The dried material was ground to a fine powder that passed through a No. 60 mesh sieve (ASTM E-11) [36,37], and stored in airtight containers protected from light and humidity until use. The moisture content of the dried plant material was 8.3 ± 0.3%, a value comparable to those reported in other studies that used *A. vera* as raw material for obtaining extracts [38].

### 4.2. Obtaining Extracts by Ultrasound-Assisted Extraction (UAE)

Ultrasound-assisted extraction was performed following previously reported protocols [27,28,39,40]. For each extraction, 50.00 g of *A. vera* dry powder was mixed with 250 mL of solvent (solid:solvent ratio 1:5 *w*/*v*). Two solvents were evaluated: distilled water (aqueous extract) and 99% ethanol (ethanolic extract). Extraction was carried out using a BOECO Ultrasonic Cleaner (BOECO, Hamburg, Germany) at 40 kHz and 480 W for 90 min at 50.0 °C, conditions kept constant for both solvents to ensure comparability. After sonication, the extracts were vacuum-filtered through Whatman No. 1 paper (Whatman, Maidstone, UK).

For the ethanolic extract, the filtrate was concentrated in an ISOLAB rotary evaporator (ISOLAB Laborgeräte GmbH, Eschau, Germany) at 50 °C, 336 mbar, and 120 rpm for 120 min, followed by oven drying (Memmert GmbH + Co. KG, Schwabach, Germany) at 50 °C for 3 days until constant weight (<0.01 g variation over 24 h). For the aqueous extract, water was first removed by rotary evaporation at 60 °C, 336 mbar, and 120 rpm for 180 min, followed by oven drying at 50 °C for 7 days until constant weight. Drying at 50 °C was selected to preserve thermolabile compounds and inhibit microbial growth, as higher temperatures (75–100 °C) degrade polysaccharides, anthraquinones, and flavonoids [34,35].

Extraction yield (%) was calculated as (weight of dry extract/weight of dry plant material) × 100. The dry extracts were resuspended in DMSO, sterilized by vacuum filtration (0.22 µm), and stored in amber flasks at 4 °C. All extractions were performed in triplicate (*n* = 3). Stock solution concentrations were verified by gravimetry: 870 mg/mL (aqueous) and 850 mg/mL (ethanolic), a difference of only 2.3%. These stock solutions were used for FTIR characterization, MIC/MBC assays, and well diffusion tests.

### 4.3. Characterization by FTIR Spectroscopy

The phytochemical characterization of the extracts was performed using Fourier transform infrared spectroscopy (FTIR) with an Attenuated Total Reflectance (ATR) accessory. An iS50 FTIR spectrometer (Thermo Fisher Scientific, Waltham, MA, USA) equipped with a diamond ATR crystal was used. The FTIR-ATR technique has proven to be an effective method for the rapid and non-destructive characterization of active compounds in medicinal plant extracts [8,13,17,22,26,41].

The identification of functional groups was performed by assigning the characteristic absorption bands: O–H stretching (3300–3400 cm^−1^) for alcohols, phenols, and polysaccharides; C–H stretching (2800–2950 cm^−1^) for aliphatic compounds; C=O/C=C stretching (1600–1650 cm^−1^) for conjugated carbonyls and unsaturated bonds potentially associated with anthraquinones and flavonoids; and C–O–C stretching (1000–1150 cm^−1^) for glycosidic bonds of polysaccharides. The spectra were processed using OMNIC software (version 9.2; Thermo Fisher Scientific, Waltham, MA, USA).

For FTIR analysis, aliquots of constant volume (approximately 2 µL) of the stock solutions of each extract in DMSO (870 mg/mL for the aqueous and 850 mg/mL for the ethanolic) were deposited directly onto the diamond crystal of the ATR accessory, configured for 32 scans, applying constant and controlled pressure using the equipment’s pressure accessory. Prior to each measurement, a spectrum of pure DMSO was recorded as a blank, which was automatically subtracted. The spectra were normalized by the total area under the curve in the range of 4000–500 cm^−1^ to allow for a semi-quantitative comparison of the relative intensities of the characteristic bands between both extracts. Since the concentrations of the stock solutions differed by only 2.3%, the comparison conditions can be considered practically equivalent.

### 4.4. Antimicrobial Activity Evaluation

The antimicrobial activity of the extracts was evaluated against the reference strains *S. aureus* ATCC 12493 (Gram-positive) and *E. coli* ATCC 33876 (Gram-negative) both originally from the American Type Culture Collection (ATCC, Manassas, VA, USA). The strains were obtained from the culture collection of the technical department of the School of Medicine of Universidad Cesar Vallejo. The strains maintained on nutrient agar at 4 °C were reactivated in Mueller–Hinton broth (MHB) and nutrient agar at 37 °C for 18–24 h prior to the assays. For inoculum preparation, isolated colonies from fresh cultures (18–24 h) were selected and suspended in sterile saline solution (0.85% NaCl) until reaching a turbidity equivalent to the 0.5 McFarland standard (approximately 1.5 × 10^8^ CFU/mL). This suspension was diluted 1:100 to obtain a final concentration of approximately 1.5 × 10^6^ CFU/mL for the assays [31,32].

#### 4.4.1. Determination of the Minimum Inhibitory Concentration (MIC) by Broth Macrodilution

The Minimum Inhibitory Concentration (MIC) was determined using the broth macrodilution method, following the standardized protocols of CLSI [32]. Serial twofold dilutions of each extract were prepared in Mueller–Hinton broth (MHB), yielding final concentrations ranging from 870.0 to 3.4 mg/mL for the aqueous extract and from 850.0 to 3.3 mg/mL for the ethanolic extract, with a final volume of 1 mL per tube. The DMSO concentrations varied from 50% (*v*/*v*) in the highest dilution to 0.2% (*v*/*v*) in the lowest, and a solvent control containing DMSO at 50% (*v*/*v*) was included. Each tube was inoculated with 100 µL of a bacterial suspension adjusted to the 0.5 McFarland standard (1.5 × 10^6^ CFU/mL), resulting in a final inoculum of approximately 1.5 × 10^5^ CFU/mL per tube. Three controls were included: (1) growth control (inoculum without extract), (2) sterility control (medium without inoculum), and (3) solvent control (DMSO at 50% *v*/*v*). Tubes were incubated at 37 °C for 18–24 h, and the MIC was defined as the lowest extract concentration that inhibited visible microbial growth. All determinations were performed in triplicate (*n* = 3).

#### 4.4.2. Determination of the Minimum Bactericidal Concentration (MBC) by Broth Macrodilution

The Minimum Bactericidal Concentration (MBC) was determined from the tubes without visible growth obtained in the MIC assay. For this, 10 μL from each tube without visible growth was plated onto Mueller–Hinton agar (MHA) plates and incubated at 37 °C for 24 h. The MBC was defined as the lowest concentration of the extract that showed no bacterial growth on the agar, i.e., the concentration that kills 99.9% of the initial inoculum [32]. All determinations were performed in triplicate (*n* = 3).

#### 4.4.3. Determination of Antimicrobial Susceptibility by the Well Diffusion Method

The antimicrobial activity of the extracts was evaluated using the agar well diffusion method, a modification of the disk diffusion technique that allows for a greater sample loading capacity. The bacterial inoculum, adjusted to 0.5 on the McFarland scale (≈1.5 × 10^8^ CFU/mL), was swabbed uniformly over the entire surface of Mueller–Hinton agar (MHA) plates. After 15 min to allow for inoculum absorption, wells of 6 mm diameter were punched in the agar using a sterile cork borer. In each well, 100 μL of the extract solutions at the evaluated concentrations was deposited; these were prepared from the stock solutions of each extract in DMSO (870 mg/mL for the aqueous and 850 mg/mL for the ethanolic) by appropriate dilutions, to obtain final concentrations of 200, 100, 50, 25, and 12.5 mg/mL. Additionally, ciprofloxacin (5 μg/well) for *E. coli* and vancomycin (30 μg/well) for *S. aureus* were used as positive controls, while the negative control consisted of the solvent (DMSO). The plates were incubated at 37 °C for 18–24 h. After incubation, the diameter of the inhibition halos (mm) around each well was measured using a Vernier caliper. Ten replicates were performed for each concentration and microorganism. It should be noted that this study was limited to in vitro assays and did not include animal models or clinical trials to evaluate the in vivo efficacy and safety of the extracts, which is recognized as a limitation and is proposed as part of future research.

### 4.5. Statistical Analysis

Data were expressed as mean ± standard deviation. Normality of the data was evaluated using the Shapiro–Wilk test, and homogeneity of variances using Levene’s test. To compare the effects of extract type (aqueous vs. ethanolic), microorganism (*S. aureus* vs. *E. coli*), and their interaction on inhibition diameters at concentrations of 200 and 100 mg/mL, a two-way factorial analysis of variance (two-way ANOVA) was performed. All statistical analyses were performed using IBM SPSS Statistics software (version 26; IBM Corp., Armonk, NY, USA), considering a significance level of *p* < 0.05. The sample size was determined based on the variability observed in previous studies of antimicrobial characterization of *A. vera* extracts [3,13,28] and on the availability of experimental resources. For the MIC/MBC assays, *n* = 3 independent replicates per treatment were used, which is consistent with CLSI recommendations for antimicrobial susceptibility testing by macrodilution [32]. For the well diffusion assays, *n* = 10 replicates per treatment were used to reduce the variability associated with manual halo measurement and to increase the statistical power of the two-way ANOVA. MIC/MBC values were reported descriptively (mean ± standard deviation), and no inferential statistical test was applied to them, given the discrete nature of twofold dilution data and the limited number of replicates.

## 5. Conclusions

The present study compared the effect of the extraction solvent on the phytochemical profile and antimicrobial efficacy of *A. vera* extracts against two reference bacterial strains. UAE and FTIR were used as methodological tools to evaluate this effect. FTIR analysis showed that the aqueous extract presented higher relative intensity in bands consistent with structural polysaccharides (O–H and C–O–C), such as acemannan and glucomannans, while the ethanolic extract showed higher relative intensity in the 1600–1650 cm^−1^ band (C=O/C=C), which may be consistent with the presence of anthraquinones and flavonoids, compounds with recognized direct antimicrobial activity. However, these FTIR differences are semi-quantitative and inconclusive regarding specific compounds, and require confirmation by complementary techniques such as HPLC-MS or NMR.

Regarding biological activity, the ethanolic extract showed a trend toward greater antimicrobial efficacy compared to the aqueous extract against the strains of *S. aureus* and *E. coli*, evidenced by significantly larger inhibition diameters in the well diffusion test and lower mean Minimum Inhibitory Concentration (MIC) and Minimum Bactericidal Concentration (MBC) values; however, no specific statistical analysis was applied to the MIC/MBC data, and these results remained inconclusive because of their high variability (*n* = 3). This trend may be related to its FTIR profile, although the compounds responsible and their mechanisms of action were not identified or evaluated in this study. Likewise, both extracts showed larger inhibition halos at 200 and 100 mg/mL against the Gram-positive strain *S. aureus* compared to the Gram-negative *E. coli*, possibly attributable to the permeability barrier represented by the outer membrane of Gram-negative bacteria.

The statistical analysis using two-way ANOVA of the inhibition halos confirmed the significance of the effects of extract type and microorganism on halo size, as well as the interaction between both factors at 100 mg/mL. These preliminary findings suggest the potential of the ethanolic *A. vera* extract as a candidate for further study against the two reference strains evaluated (*S. aureus* ATCC 12493 and *E. coli* ATCC 33876); these results cannot be extrapolated to other microorganisms, to a broad antimicrobial spectrum, or to resistant clinical strains, which were not evaluated.

From an applied perspective, and within the limits of this preliminary study, 99% ethanol appears to be a suitable solvent for obtaining extracts with greater activity against the two reference strains evaluated, while water may be more appropriate for the recovery of hydrophilic components, such as polysaccharides. FTIR-ATR analysis may serve as a complementary tool for monitoring extract consistency, and the standardization of extraction conditions would improve reproducibility between batches. Any potential antimicrobial or therapeutic application of these extracts would require additional studies to validate their efficacy, safety (including cytotoxicity), and reproducibility.

The study presents some limitations, including the phytochemical characterization based solely on FTIR, the antimicrobial evaluation limited to two reference strains, the high variability of the MIC/MBC results obtained with a limited number of replicates, and the absence of evaluation against resistant clinical strains and of cytotoxicity, biocompatibility, and in vivo efficacy assays. Future research should identify and quantify the bioactive compounds using more specific analytical techniques, confirm the MIC/MBC results by broth microdilution with a larger number of replicates, expand the evaluation to clinical isolates and multidrug-resistant strains, and conduct safety and efficacy studies. These analyses will allow for a more precise assessment of the potential of *A. vera* extracts.

## Figures and Tables

**Figure 1:**
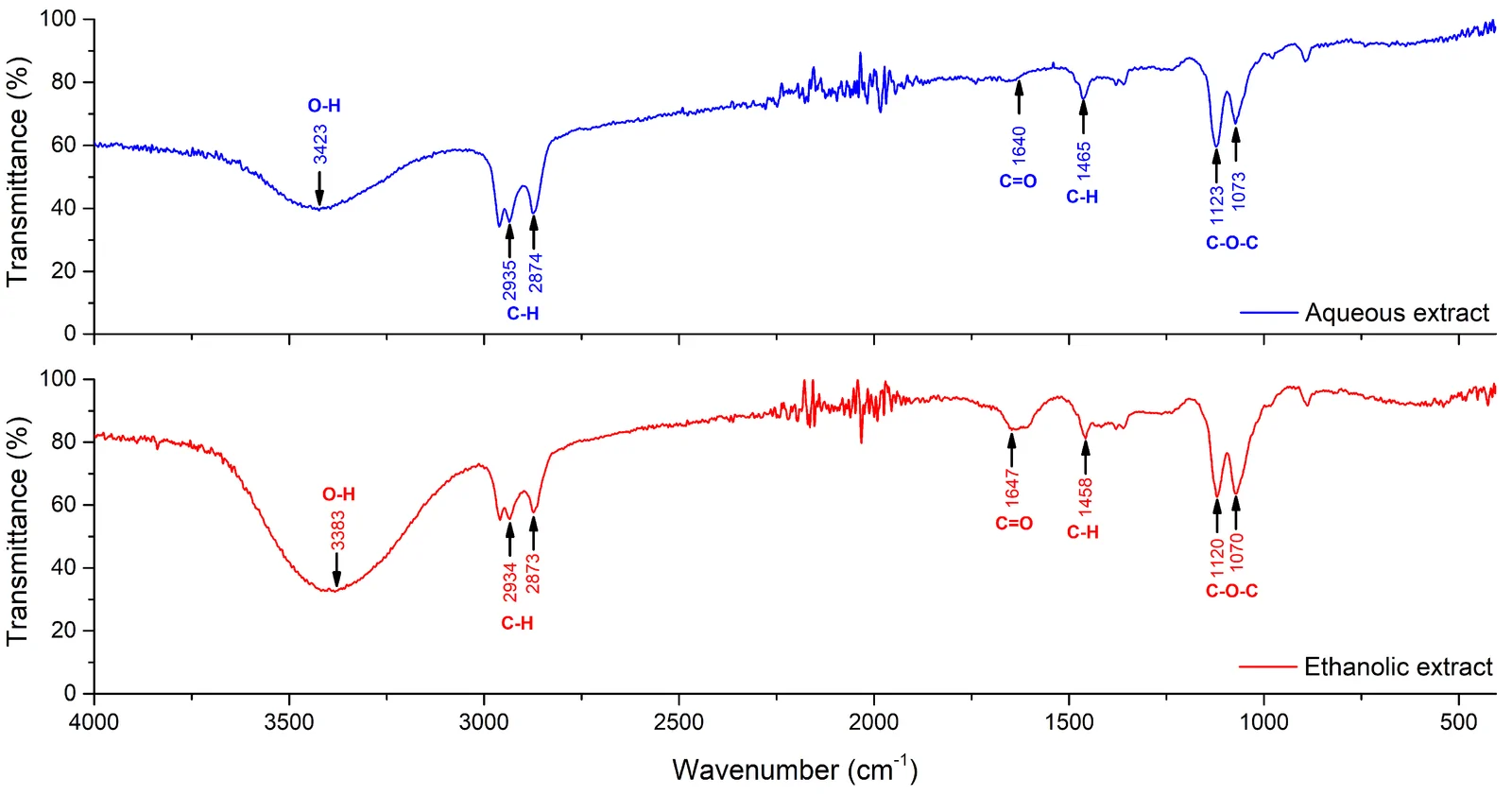
FTIR spectra of the aqueous extract (upper panel, blue line) and the ethanolic extract (lower panel, red line) of *Aloe vera* obtained by ultrasound-assisted extraction (UAE), recorded in the range of 4000–500 cm^−1^. Arrows and labels on the curves indicate the key differential bands: O–H (3300–3400 cm^−1^), C–H (2800–2950 cm^−1^), C=O/C=C (1600–1650 cm^−1^), and C–O–C (1000–1150 cm^−1^).

**Table 1:** Extraction yield of aqueous and ethanolic *Aloe vera* extracts obtained by ultrasound-assisted extraction (UAE).

Extraction Solvent	Plant Part	Initial Weight (g)	Dry Extract Weight (g)	Extraction Yield (% *w*/*w*) *
Water	Leaves (rind, gum, and pulp)	50.00	7.03 ± 0.15	14.13 ± 0.31
99% Ethanol	Leaves (rind, gum, and pulp)	50.00	6.40 ± 0.16	12.80 ± 0.32

* Values represent the mean ± standard deviation (*n* = 3).

**Table 2:** Phytochemical characterization by FTIR, relative abundance of functional groups, and proposed antimicrobial mechanisms for *A. vera* extracts.

Range (cm^−1^)	Functional Group	Extract with Higher Intensity	Interpretation and Proposed Antimicrobial Mechanism *
3300–3400	O–H (stretching)	Aqueous	The aqueous extract shows higher relative intensity, consistent with the presence of polysaccharides (acemannan, glucomannans) and phenolic compounds. The hydroxyl groups of *A. vera* phenolic compounds can directly interact with the bacterial cell wall through hydrogen bonds, altering its structural integrity and intrinsic permeability [8,17].
2800–2950	C–H (stretching)	Similar intensity in both extracts	Similar intensity in both extracts, indicating a comparable presence of lipid and aliphatic compounds. These compounds can complementarily contribute to the disruption of the bacterial envelope by physically intercalating into the lipid bilayer, increasing cell fluidity and destabilizing its hydrophobic organization [26].
1600–1650	C=O /C=C (stretching)	Ethanolic	The ethanolic extract shows higher relative intensity, a spectral feature potentially associated with anthraquinones and flavonoids, compounds with recognized antimicrobial activity. These compounds exert direct microbicidal action through membrane disruption, inhibition of DNA/protein synthesis, and interference with solute transport [8,17,26].
1000–1150	C–O–C (stretching)	Aqueous	The aqueous extract shows higher relative intensity, consistent with structural polysaccharides such as acemannan and glucomannans (major components in *A. vera*). The major gel polysaccharides exert antimicrobial effects through: (1) modulation of the immune response; (2) formation of a physical barrier that prevents bacterial adhesion; and (3) bacteriostatic activity through interference in the biosynthesis pathways of bacterial cell wall components [5,9].

* Band assignments correspond to functional groups and compound families associated according to the literature. It should be noted that the absorption band around 1600–1650 cm^−1^ may be attributed to both conjugated carbonyl groups (C=O) and carbon–carbon double bonds (C=C), and FTIR alone cannot distinguish between these possibilities. FTIR analysis does not allow for the unequivocal identification of individual compounds; confirmation of specific compounds requires complementary techniques such as HPLC-MS or NMR. The proposed mechanisms are based on literature reports for compounds with similar functional groups and have not been experimentally evaluated in the present study.

**Table 3:** Minimum Inhibitory Concentration (MIC) and Minimum Bactericidal Concentration (MBC) of aqueous and ethanolic *Aloe vera* extracts against reference bacterial strains.

Extract Type	Microorganism Evaluated	MIC (mg/mL)	MBC (mg/mL)
Aqueous	*S. aureus* ATCC 12493	90.7 ± 31.4	145.0 ± 62.8
	*E. coli* ATCC 33876	145.0 ± 62.8	181.3 ± 62.8
Ethanolic	*S. aureus* ATCC 12493	62.0 ± 40.6	106.3 ± 0.0
	*E. coli* ATCC 33876	85.6 ± 30.7	177.1 ± 61.3

MIC = Minimum Inhibitory Concentration; MBC = Minimum Bactericidal Concentration. Values represent the mean ± standard deviation (*n* = 3).

**Table 4:** Inhibition diameters (mm) of aqueous and ethanolic *Aloe vera* extracts against *S. aureus* ATCC 12493 and *E. coli* ATCC 33876 at different concentrations.

Extract Type	Microorganism Evaluated	Inhibition Halos (mm) by Concentration				
		200 mg/mL	100 mg/mL	50 mg/mL	25 mg/mL	12.5 mg/mL
Aqueous	*S. aureus*	20.6 ± 0.4	13.6 ± 0.5	0.0	0.0	0.0
	*E. coli*	17.4 ± 0.5	11.6 ± 0.6	0.0	0.0	0.0
Ethanolic	*S. aureus*	22.3 ± 0.5	14.3 ± 0.5	8.1 ± 0.6	0.0	0.0
	*E. coli*	20.0 ± 0.4	13.8 ± 0.6	8.5 ± 0.6	0.0	0.0

Note: Positive controls were vancomycin (30 µg/well) against *S. aureus* ATCC 12493, with an inhibition halo of 30.0 ± 0.6 mm, and ciprofloxacin (5 µg/well) against *E. coli* ATCC 33876, with a halo of 25.0 ± 0.6 mm (*n* = 10). The negative control (DMSO) did not produce an inhibition halo (0.0 mm) in any case.

**Table 5:** Results of the statistical analysis (two-way ANOVA) of the inhibition halos of aqueous and ethanolic *Aloe vera* extracts against *S. aureus* ATCC 12493 and *E. coli* ATCC 33876.

Concentration (mg/mL)	Extract	*S. aureus* ATCC 12493	*E. coli* ATCC 33876	*p*-Value (Extract)	*p*-Value (Microorg.)	*p*-Value (Interaction)
200	Aqueous	20.57 ± 0.43	17.37 ± 0.47	<0.001	<0.001	0.138
	Ethanolic	22.33 ± 0.49	20.02 ± 0.42			
100	Aqueous	13.64 ± 0.46	11.61 ± 0.58	<0.001	<0.001	<0.001
	Ethanolic	14.27 ± 0.45	13.81 ± 0.59			
50	Aqueous	0.00 ± 0.00	0.00 ± 0.00	N/A	N/A	N/A
	Ethanolic	8.11 ± 0.58	8.54 ± 0.57			
25	Aqueous	0.00 ± 0.00	0.00 ± 0.00	N/A	N/A	N/A
	Ethanolic	0.00 ± 0.00	0.00 ± 0.00			

## Data Availability

The original contributions presented in this study are included in the article.
